# Experiences of informational needs and received information following a prenatal diagnosis of congenital heart defect

**DOI:** 10.1002/pd.4815

**Published:** 2016-04-24

**Authors:** Tommy Carlsson, Gunnar Bergman, Barbro Wadensten, Elisabet Mattsson

**Affiliations:** ^1^ Department of Public Health and Caring SciencesUppsala UniversityUppsalaSweden; ^2^ Department of Women's and Children's HealthKarolinska InstitutetStockholmSweden; ^3^ Department of Women's and Children's HealthUppsala UniversityUppsalaSweden

## Abstract

**Objective:**

To explore the need for information and what information was actually received following prenatal diagnosis of a congenital heart defect, in a country where termination of pregnancy beyond 22 weeks of gestation is not easily possible because of legal constraints.

**Methods:**

Twenty‐six Swedish‐speaking pregnant women (*n* = 14) and partners (*n* = 12) were consecutively recruited for semi‐structured telephone interviews following the prenatal diagnosis of a congenital heart defect. Data were analyzed using content analysis.

**Results:**

Although high satisfaction with the specialist information was described, the information was considered overwhelming and complex. Objective, honest, and detailed information about multiple subjects were needed, delivered repeatedly, and supplemented by written information/illustrations. Eighteen respondents had used the Internet to search for information and identified issues involving searching difficulties, low quality, and that it was too complex, insufficient, or unspecific. Those who terminated their pregnancy criticized that there was a lack of information about termination of pregnancy, both from health professionals and online sources, resulting in unanswered questions and unpreparedness.

**Conclusion:**

Individuals faced with a prenatal diagnosis of a congenital heart defect need individualized and repeated information. These needs are not all adequately met, as individuals are satisfied with the specialist consultation but left with unanswered questions regarding pregnancy termination. © 2016 The Authors. *Prenatal Diagnosis* published by John Wiley & Sons, Ltd.

## Abstract 2

**What's Already Known About This Topic?**

- The introduction of ultrasound screening during the second trimester of pregnancy has increased the detection rate of major congenital heart defects.
- Information matched to individual needs is crucial for women to cope with the situation following a prenatal diagnosis.

**What Does This Study Add?**

- There is a need for information on multiple subjects following the prenatal diagnosis of congenital heart defect, including termination of pregnancy.
- Although information is available on the Internet on congenital heart defects, it is not easily searchable and its quality may be less than desired.
- Apparently, there is insufficient information about termination of pregnancy following the prenatal diagnosis of congenital heart defect.

## Introduction

Prenatal screening during the second trimester of pregnancy has greatly improved the detection rate of major congenital heart defects (CHD).^1^ Pregnant women have optimistic expectations on the scan, viewing it as a confirmation of their expected child to be healthy.^2^, ^3^ Thus, they are unprepared for a prenatal diagnosis of a congenital birth defect,^2^, ^3^, ^4^, ^5^, ^6^ which results in acute grief reactions^7^ and psychological distress.^7^, ^8^, ^9^

Depending on state laws, termination of pregnancy is an option following that detection. The decision involves ethical dilemmas^10^ and informational difficulties.^9^, ^11^, ^12^ The decision regarding the future of the pregnancy entails a process of chosen loss and lost choices, as the pregnancy is wanted but the fetal malformation is not.^6^ Information matched to individual needs is crucial for women to cope with the situation following a prenatal diagnosis,^4^, ^13^ and it has previously been concluded that expecting parents consistently rank informational subjects as more important than pediatric cardiologists do.^14^ Women faced with a prenatal diagnosis of a malformation have variable informational needs,^13^ which occasionally are unmet.^11^, ^13^ This raises concerns about clinical practice regarding information coverage and delivery. Furthermore, the Internet is used to supplement information from the specialist,^12^ but this involves searching difficulties, quality deficits,^15^ and an overwhelming amount of available information.^12^

To reach conclusions regarding specific informational needs following a prenatal diagnosis of CHD, inductive research is required to provide a foundation for clinical practice. Thus, the aim of this study was to explore the need for information and what information was actually received following a prenatal diagnosis of a congenital heart defect, in a country where termination of pregnancy beyond 22 weeks of gestation is virtually not performed because of legal constraints.

## Methods

### Setting

The study was performed at two tertiary referral centers for fetal cardiology and fetal medicine at Uppsala University Hospital, Uppsala and Astrid Lindgren Children's Hospital, Stockholm, Sweden. In Sweden, all pregnant women are offered ultrasound screening at approximately 18 weeks of gestation. Suspected heart malformations are referred to a fetal cardiologist for a specialist consultation. Based on the findings and precision of the ultrasound examination, oral information is offered on a broad variety of topics, in addition to drawings of the heart defect. The risk of associated malformations and chromosomal abnormalities is highlighted, and additional fetal medical investigations are often offered through close cooperation with the fetal medicine unit. Following the diagnosis made by the fetal cardiologist, pregnant women are presented with the option of choosing termination of pregnancy prior to a gestational age of 18 weeks and 0 days, and later after approval from the National Board of Health and Welfare, as stated in the Swedish Abortion Act.^16^ In clinical practice, approval is seldom given after 22 gestational weeks.

If the woman decides to terminate the pregnancy, a social worker assists her with the application to the National Board of Health and Welfare and offers professional psychosocial support. Follow‐up visits are offered at the fetal medicine unit and, if needed, also at the fetal cardiology unit.

When pregnancy is continued, fetal cardiology follow‐up visits are offered every 4 to 6 weeks in addition to the routine prenatal care, to monitor the progression of the CHD, prepare the couple and to optimize the planning of prenatal management. If needed, the expectant parents are offered professional psychosocial support by a social worker at the unit.

### Recruitment

To be considered for inclusion, potential participants needed to speak Swedish and be either pregnant with a fetus diagnosed with a CHD before 22 gestational weeks or the partner of such a pregnant woman. Between March and November 2014, consecutive^17^ pregnant women and their partner were invited to participate by a pediatric nurse. Oral and written information about the study were given after the initial fetal cardiology consultation. Potential participants were informed that participation in the study was entirely voluntary and that their decision to participate or not would not affect future care. If the potential participants needed more time to deliberate about whether they wanted to participate, the pediatric nurse contacted them later via telephone to collect decline/consent. All who agreed to participate completed a written informed‐consent form and were contacted by the first author 5 to 15 weeks after inclusion. This timeframe was chosen to give the respondents some time to reflect on their situation while still retaining memories with enough detail to answer the specific questions.

**Figure 1:**
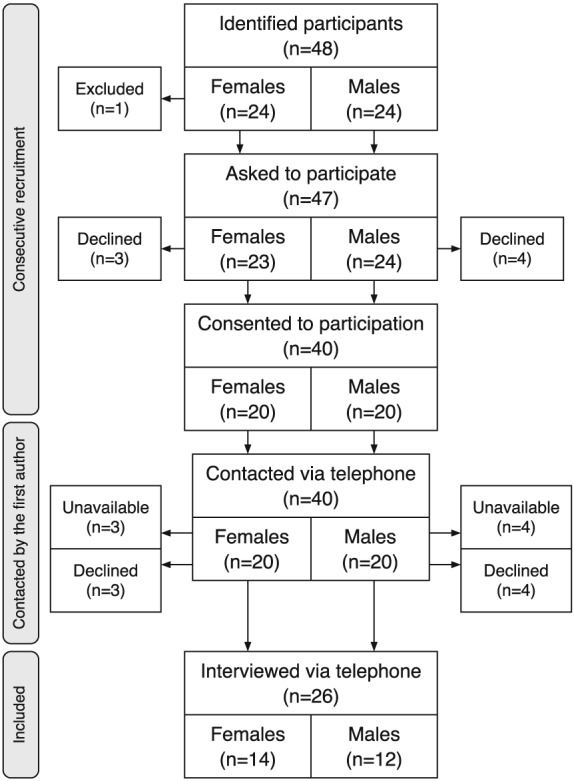
The recruitment process

Figure 1 presents the recruitment process. During the inclusion period, 48 potential participants were identified; one woman was excluded because of language difficulties, leaving 23 pregnant women and 24 male partners. Of these, 40 consented and were contacted via telephone. However, seven participants were unavailable when called (three women and four men), and seven declined participation (three women and four men) because of personal reasons (*n* = 4), no interest (*n* = 2), and lack of time (*n* = 1). Thus, 26 participants, 14 women, and 12 men, were interviewed via telephone (65% of consented participants). In ten cases, both the woman and her partner were interviewed; in four cases, only the woman was interviewed, and in two cases, only the male partner was interviewed.

### Data collection

The interview took place 6 to 15 weeks after the diagnosis (mean = 12 weeks). The mean interview duration was 31 min among those who continued the pregnancy (range = 21–47) and 44 min among those who terminated the pregnancy (range = 20–89). The interviews were digitally recorded and transcribed verbatim by the first author. Handwritten notes were kept during the course of the interviews.

At the beginning of the interview, the respondents were asked structured questions about their age, highest educational level, number of children, country of birth, termination/continuation of pregnancy, and associated anomalies. The type of heart defect and gestational age at diagnosis were collected from the medical record. An interview guide was used to stay relevant to the aim^18^ (Table S1 for the interview guide). Additionally, the interview contained questions about the decisional process and emotional situation. However, the findings of these two research questions will not be presented here. Probes such as ‘Can you tell me more, please?’ were used to explore further. At the end of the interview, a summary was made and the participants were asked if they wanted to correct or add anything. Data collection and analysis were conducted concurrently and carried out until data saturation was achieved, that is, further data collection and analysis did not deepen or challenge the findings.^18^

### Data analysis

Utilizing Nvivo for Mac version 10.2.0 (QSR International, Doncaster, Victoria, Australia), the first author analyzed the texts using inductive qualitative content analysis.^19^ After reading the transcripts repeatedly, meaning units were identified, defined as words, sentences or paragraphs containing aspects related to each other through their content and context.^19^ These were condensed and assigned a descriptive code, which were structured into categories and subcategories, that is, collections of different codes that share commonality of content. The categories represent the manifest content, that is, the visible and obvious components in the data. One theme was formed from the categories, defined as the underlying meaning and representing the interpreted latent content. This analysis moved back and forth dialectically, and discussions were held with the last author until consensus was reached.

To gain insight into preconceptions, the first author kept reflective notes during the course of the study. The first author is a specialist nurse and PhD student with no personal experience with fetal defects or parenthood. He does not have any professional experience with prenatal care. However, he has previously conducted research interviews with parents with experience of a prenatal diagnosis,^12^ assessed quality of websites about prenatally detected CHD,^15^ and explored virtual community threads about prenatal diagnosis (unpublished data).

### Ethical considerations

The study was approved by the Regional Ethics Committee in Uppsala and Stockholm, Sweden.

## Results

### Population characteristics

Table 1 presents characteristics of the included participants who continued (*n* = 11) and terminated the pregnancy (*n* = 15) with distributions of women and men. In summary, the participants who decided to terminate the pregnancy had a univentricular diagnosis or associated chromosomal and extracardiac anomalies, Table 2.

**Table 1:** Characteristics of included participants (*n* = 26) who continued (*n* = 11) and terminated the pregnancy (*n* = 15) with distributions of women (*n* = 14) and men (*n* = 12)

Characteristic	Category	Continued pregnancy (*n* = 11)		Terminated pregnancy, (*n* = 15)		Total, *n* (%)
		Women (*n* = 6), *n* (%)	Men (*n* = 5), *n* (%)	Women (*n* = 8), *n* (%)	Men (*n* = 7), *n* (%)	
Participant age	20–29	1 (3.8)	1 (3.8)	5 (19.2)	2 (7.7)	9 (34.6)
	30–39	4 (15.4)	3 (11.5)	2 (7.7)	3 (11.5)	12 (46.2)
	>39	1 (3.8)	1 (3.8)	1 (3.8)	2 (7.7)	5 (19.2)
Number of children	0	2 (7.7)	2 (7.7)	5 (19.2)	2 (7.7)	11 (42.3)
	1	4 (15.4)	3 (11.5)	3 (11.5)	4 (15.4)	14 (53.8)
	2	0 (0.0)	0 (0.0)	0 (0.0)	1 (3.8)	1 (3.8)
Highest education	Senior high school	2 (7.7)	2 (7.7)	2 (7.7)	3 (11.5)	9 (34.6)
	University/College	4 (15.4)	3 (11.5)	6 (23.1)	4 (15.4)	17 (65.4)
Participant country of birth	Sweden	6 (23.1)	5 (19.2)	8 (30.8)	5 (19.2)	24 (92.3)
	Other	0 (0.0)	0 (0.0)	0 (0.0)	2 (7.7)	2 (7.7)
Gestational week at diagnosis	17	0 (0.0)	0 (0.0)	0 (0.0)	1 (3.8)	1 (3.8)
	18	1 (3.8)	1 (3.8)	4 (15.4)	3 (11.5)	9 (34.6)
	19	2 (7.7)	1 (3.8)	1 (3.8)	1 (3.8)	5 (19.2)
	20	3 (11.5)	3 (11.5)	3 (11.5)	2 (7.7)	11 (42.3)

**Table 2:** Experienced fetal diagnoses among the participants (*n* = 26) who continued (*n* = 11) and terminated the pregnancy (*n* = 15) with distributions of women (*n* = 14) and men (*n* = 12)

Diagnosis	Continued pregnancy (*n* = 11)		Terminated pregnancy, (*n* = 15)		Total, *n* (%)
	Women (*n* = 6), *n* (%)	Men (*n* = 5), *n* (%)	Women (*n* = 8), n (%)	Men (*n* = 7), *n* (%)	
Univentricular	2 (7.7)	1 (3.8)	6 (23.1)	4 (15.4)	13 (50.0)
Hypoplastic left heart syndrome	1 (3.8)	0 (0.0)	1 (3.8)	0 (0.0)	2 (7.7)
Critical aortic stenosis	0 (0.0)	0 (0.0)	2 (7.7)	2 (7.7)	4 (15.4)
Double inlet left ventricle	0 (0.0)	0 (0.0)	2 (7.7)	1 (3.8)	3 (11.5)
Tricuspid atresia	1 (3.8)	1 (3.8)	1 (3.8)	1 (3.8)	4 (15.4)
Tetralogy of fallot	3 (11.5)	3 (11.5)	0 (0.0)	0 (0.0)	6 (23.1)
Atrioventricular septal defect in association with trisomy 21	0 (0.0)	0 (0.0)	1 (3.8)	2 (7.7)	3 (11.5)
Coarctation of aorta	1 (3.8)	1 (3.8)	0 (0.0)	0 (0.0)	2 (7.7)
Ebstein's anomaly/tricuspid dysplasia associated with multiple extracardial structural malformations	0 (0.0)	0 (0.0)	1 (3.8)	0 (0.0)	1 (3.8)
Pulmonary atresia with ventricular septal defect and major aortopulmonary collateral arteries	0 (0.0)	0 (0.0)	0 (0.0)	1 (3.8)	1 (3.8)

### Overarching theme: hunting for information in a confusing reality

The analysis resulted in the overarching theme ‘Hunting for information in a confusing reality’, including six categories.

#### Category 1: informational methods (Table 3)

**Table 3:** Identified needs for informational methods with illustrative quotes and number of respondents (*n* = 26) who mentioned a need

Informational method needs	Illustrative quote	*n*
Illustrations	*Be a little pedagogic as well*, *draw a heart on a piece of paper and explain how the heart works*… (*Male 7*, *CP*)	*17*
Detailed	*You want to know everything in the greatest detail*. (*Male 10*, *TP*)	*16*
Written	*This need for a brochure or an Internet site or something where you can go back and read*… (*Female 9*)	*13*
Early	*Of course you want to find out about it as early as possible*… (*Male 7*, *CP*)	*9*
Objective	*If someone had been for it* [*one of the alternatives*]… *then you couldn*'*t have made a choice*. (*Female 4*, *TP*)	*8*
Repeated	*Then we met them several times as well*, *so then we remembered everything*. (*Female 3*, *TP*)	*8*
Specialist information	*You want to have information from so close to the source as possible* (*Male 6*, *CP*)	*7*
Honest	*It*'*s better that they say precisely what they think it is rather than not saying anything*. (*Female 4*, *TP*)	*6*
Available	*Though we got something which made things easier I think*, *we got a direct line to everyone*. (*Female 3*, *TP*)	*4*
Mother tongue	*What could have been better*, *that we could have got it* [*an illustration*] *in Swedish*. (*Female 7*, *CP*)	*2*

CP, continued pregnancy; TP, terminated pregnancy.

The respondents needed objective, honest, and detailed information delivered repeatedly. They valued early and available specialist information, supplemented by written information, and illustrations (Table S2 for an expanded presentation of the findings).

#### Category 2: informational content (Table 4)

**Table 4:** Identified needs for informational content with illustrative quotes and number of respondents (*n* = 26) who mentioned a need

Content	Illustrative quote	*n*
Postnatal situation		26
Quality of life for child	That it can go and have a good life. (*Female 8, CP*)	18
Treatments	*If it was somehow possible to operate*. (*Female 11*, *TP*)	17
Mortality	*If he could live afterwards*. (*Female 4*, *TP*)	9
Prognosis	*What is the prognosis*? (*Male 12*, *TP*)	7
Practical issues	*You can*'*t go in the same transportation*. (*Female 2*, *CP*)	5
Possible complications	*What sort of problems the heart defect could entail*. (*Female 7*)	4
Activity	*If she could go out in the woods with us*. (*Male 6*)	3
Family life	*For the whole family like*. (*Female 8*)	2
Follow‐up care	*Very important to know what the chain will look like*. (*Male 1*)	1
School	*If she could go to school as normal*. (*Male 6*, *CP*)	1
Previous cases	*Listen to other people who have gone through exactly the same thing is quite*… *I like that*. (*Male 11*, *CP*)	18
Termination of pregnancy		16
The option	*What choices you can make*. (*Male 11*, *CP*)	13
The abortion procedure	*It can be extra important that they explain how a termination is done*. (*Female 6*, *TP*)	7
Fetal care	*I would gladly have got a bit more information about how the fetus is taken care of*. (*Female 6*, *TP*)	3
Fetal status	*Does the child suffer*… (*Female 12*, *TP*)	3
Decision to see the fetus	*When the fetus has come out*, *you will have the chance to*, *yes*, *see the child or fetus*. (*Male 8*, *TP*)	2
Recuperation	*Can I work out again*, *is it OK*? (*Female 3*, *TP*)	2
Time required	*It might have been better if we were told that it would be such a long time*… (*Male 3*, *TP*)	2
Anatomy	*What the actual heart defect looked like*. (*Female 7*, *CP*)	11
Associated anomalies	*It was all that about Down*'*s syndrome*. (*Male 8*, *TP*)	8
Statistics	*In a perfect world or what should I say*, *I would have liked statistics*. (*Female 6*, *TP*)	7
Causes	*Is it our lifestyle or what we have done*. (*Male 5*, *TP*)	3
Professional psychosocial support	*She asked me why don*'*t you talk to someone in the meantime*, *and I just*, *I didn*'*t know that it was even possible*. (*Female 6*, *TP*)	1

CP, continued pregnancy; TP, terminated pregnancy.

The respondents mentioned multiple informational content needs. The postnatal situation of the child was mentioned by all respondents. Respondents who terminated the pregnancy described information needs concerning several aspects of termination of pregnancy, which were not mentioned by respondents who continued the pregnancy (Table S3 for an expanded presentation of the findings).

#### Category 3: a confusing situation because of unspecific initial information

The initial detection at the ultrasound scan had been completely unexpected, resulting in emotional shock. This emotional shock made it difficult to comprehend and understand the situation. Furthermore, because of unspecific and vague information at the ultrasound scan, many unanswered questions and worries remained afterwards.

*It was forty‐eight very long hours that we had to wait in a state of uncertainty*. (*Male 2*, *Termination of pregnancy*)

#### Category 4: specific information from a specialist clarifies the situation

Overall, the respondents had positive experiences of the information provided by the specialist, described as being of high quality. Valued as a definitive diagnosis from a medical expert, the respondents described that the consultation clarified their situation and brought specific information about the diagnosis. While the wait for the specialist consultation had been difficult, it also served as an opportunity to process thoughts and prepare for the consultation.

*So it was in a way good to go there*, *because we then got more information about what it was and how things would go*, *um*… *It was*, *it was quite good*. (*Female 7*, *Continuation of pregnancy*)

Occasionally though, the information provided during the specialist consultation was considered overwhelming and complex. Because of all the uncontrollable thoughts and emotions present at the consultation, respondents experienced difficulties remembering the information afterwards.

*I think I was so shocked about being told that something was wrong with the heart that I just can*'*t remember a lot of what he said like*. (*Female 6*, *Termination of pregnancy*)

#### Category 5: supplemental online information

Eighteen respondents used the Internet to search for trustworthy supplemental information, while eight avoided the Internet. Reasons for avoiding included difficulties assessing the relevance/accuracy of the information, lack of trustworthy websites, easy to focus on the worst cases, and lack of energy. Among those who searched for online information, nine reported that the search for trustworthy and high quality websites had been difficult. However, two respondents who had received advice on appropriate websites and search terms described that their searches had been easy.

*But then*, *of course*, *I went home and began*, *began searching on the Internet and tried to understand a bit more about what it was and so on*… (*Female 10*, *Continuation of pregnancy*)

Issues with the information found online involved low quality, and that it was too complex, insufficient or unspecific. Among respondents who terminated the pregnancy, websites were criticized for belittling and lacking information about the termination procedure. One respondent experienced that information websites from patient associations had been biased toward continuation of pregnancy.

*They came across as being fairly positive and with a nice tone that it will be OK in the end*. *But you could read between the lines that there were in fact problems*. (*Male 12*, *Termination of pregnancy*)

The respondents also used the Internet to read stories from others via virtual communities and blogs, which were criticized for providing unreliable and biased information. While described as valuable and comforting to read the stories, it had simultaneously been emotionally difficult and evoked fear.

*Many people wrote that it was the worst thing they had gone through in their life*. *Having to give birth to their dead baby*. *Um*… *so it was very tough as it was something that I personally thought a lot about* […] *On the other had what gave me a little strength in these forums was the fact that many people wrote that*… *Yes*, *it was a tough thing to go through*, *they will always remember it*, *but many had also decided to move on and try again*. (*Female 6*, *Termination of pregnancy*)

#### Category 6: insufficient information about termination of pregnancy

Eight respondents who terminated the pregnancy described insufficient information about termination of pregnancy, both from health professionals and trustworthy online sources. This resulted in unanswered questions and unpreparedness for the appointment, for example regarding the length of the abortion, the fact that it was an induced labor and delivery, and whether it was possible to see/hold the fetus. Three respondents, however, were content with the amount of information about the abortion and had received enough information to feel prepared for the appointment.

*I felt a bit that that information*, *that I wanted that information about how a late abortion is done*. *I would have liked to have had a little more information earlier*. *And I don*'*t think that I really got it until the day it was to be done*. *And we*'*re all different of course*, *but I thought it was really tough not really knowing what I could expect*. (*Female 3*, *Termination of pregnancy*)

## Discussion

### Principal results

Following a prenatal diagnosis of CHD, specialist consultations are necessary to offer detailed information about a number of topics, including the expected health impact on the future child and its family, and termination of pregnancy. A need for multiple informational methods was expressed, for example, repeated specialist information with supplemental written information and illustrations. The Internet was used for additional information about CHD, but problems finding high‐quality relevant and reliable websites were described. A highlight of this study is that insufficient information about pregnancy termination resulted in unanswered questions and unpreparedness.

In this study, respondents who terminated the pregnancy described unmet informational needs about the termination process. The information was described as insufficient, both from health professionals and online sources. The latter is in line with findings in a previous study investigating content and quality on publicly available information websites about CHD following a prenatal diagnosis, showing that less than 10% of websites include information about termination of pregnancy.^15^ Termination carries a social stigma,^20^ independent of state laws,^21^ and it is possible that this influences information content. Findings from this study indicate a need for clinical guidelines regarding informational responsibilities for this patient population. Traditionally in Sweden, physicians have taken on the responsibility of counseling about termination of pregnancy. However, the findings of this study indicate that additional methods to offer information are necessary to adequately meet informational needs among pregnant women and their partners choosing to terminate the pregnancy following a prenatal diagnosis. One option would be to adopt a team‐based strategy, for example midwife‐led counseling as a complement to physician information. Midwife‐led services have been found to be effective, efficient, and patient‐focused and may ease patient pressure on obstetric and pediatric cardiology specialists.^22^

In accordance with previous research,^12^, ^23^ this study observed high satisfaction with the specialist consultation. However, the information was also described as being overwhelming, complex, and insufficient. Furthermore, information from the specialist consultation was easily forgotten and supplemental written information was needed. It has previously been put forward that human memory has a limited capability and memory decays fast. Consequently, repetition of the provided information results in a more powerful imprint in long‐term memory.^24^ Previous studies have concluded that supplemental computerized learning aids,^25^ illustrations, and written information increase satisfaction and knowledge^26^ and are desired following a prenatal diagnosis.^3^, ^11^, ^12^ Taken together, the findings underscore the importance to offer repeated and supplemental information following a prenatal diagnosis of CHD.

The respondents used the Internet to search for supplemental information. As reported elsewhere,^12^, ^15^ searching difficulties on the Internet were described. However, those given advice on appropriate websites and from appropriate search terms described fewer issues. It has previously been concluded that parents of children with CHD rank information about websites significantly higher than cardiologists,^14^ and still a minority of parents receive recommendations about appropriate websites.^27^ Furthermore, it has been concluded that websites about CHD have quality and content deficits,^15^ also described in this study. Thus, health professionals should strive to recommend and provide supplemental online information when desired.

### Strengths and limitations

This study included 26 pregnant women or male partners, both those who decided to continue and those who decided to terminate the pregnancy. Because the aim was to qualitatively explore experiences, we collected data until saturation was achieved. This way, richness of the material, rather than the actual number of participants, determined the final sample size.^18^ Furthermore, the respondents were recruited consecutively via two tertiary units, which would indicate that the sample corresponds to different clinical settings. The sample is varied with regard to age and sex, but not with regard to educational level, country of birth, and associated anomalies. It is possible that the findings do not completely reflect the experiences of immigrants, individuals with low educational levels, and cases with associated anomalies. This needs to be taken into consideration when interpreting the transferability of the findings.

Previously reported possible advantages of telephone interviews in qualitative studies include practical issues, increased anonymity, decreased social pressure,^28^, ^29^, ^30^ and that respondents may feel more relaxed.^28^, ^29^, ^30^, ^31^ Potential disadvantages include decreased opportunity to create interview ambiance, reduction of social cues^29^ and lack of nonverbal communication.^30^ While telephone interviews have been reported to be shorter and include fewer details,^32^ others have reported that they produce similar material to face‐to‐face interviews.^30^ We found that the respondents valued the practical advantages of the chosen method. Offering telephone interviews made recruitment easier, and we believe that the method encouraged the participants to freely describe their experiences. However, we cannot disregard the risk that the chosen method might have produced less rich material than face‐to‐face interviews.

In qualitative studies, the researcher is the instrument for analysis.^18^ The first author conducted the primary analysis and thus influenced the findings. To tackle this, reflective notes were kept so as to be attentive to preconceptions about the phenomenon under study, and the last author took part in the later stages of analysis. Still, the analysis is a result of individual interpretations within the research group, which should be taken into consideration when interpreting the findings.

### Suggestions for future research

The findings suggest a need to further investigate satisfaction with information following a prenatal diagnosis of CHD, especially information about termination of pregnancy and preparedness for the termination procedure. Future research should investigate midwifery‐led counseling and the effect of trustworthy supplemental online information sources. Furthermore, future studies should also explore experiences among individuals with a different country of birth and low educational levels.

## Conclusion

Individuals faced with a prenatal diagnosis of a congenital heart defect need individualized and repeated information. These needs are not all adequately met, as individuals are satisfied with the specialist consultation but criticize a lack of information about termination of pregnancy and are left with unanswered questions about the abortion when deciding to terminate the pregnancy. The Internet may serve as an additional source of information following a prenatal diagnosis of CHD, but issues of finding relevant and reliable high‐quality websites exist. Furthermore, the fact that difficulties in remembering information from the specialist consultation are experienced is an incentive to investigate how trustworthy supplemental information can be adequately offered following a prenatal diagnosis of CHD.

## Supporting information

Interview guide

Identified needs for informational methods with illustrative quotes

Identified needs for informational content with illustrative quotes

## References

[1] Bergman G , Borgström E , Lundell B , Sonesson SE . Förbättrad prenatal diagnostik av medfödda hjärtfel: Uppföljningsstudie av fosterekokardiografiska undersökningar [Improved prenatal diagnosis of congenital heart defects: A follow‐up study of prenatal ultrasound screening]. Lakartidningen 2008;105:899–903. 18461855

[2] Garcia J , Bricker L , Henderson J , *et al.* Women's views of pregnancy ultrasound: A systematic review. Birth 2002;29:225–50. 12431263 10.1046/j.1523-536x.2002.00198.x

[3] Lalor JG , Devane D , Begley CM . Unexpected diagnosis of fetal abnormality: Women's encounters with caregivers. Birth 2007;34:80–8. 17324182 10.1111/j.1523-536X.2006.00148.x

[4] McCoyd JL . What do women want? Experiences and reflections of women after prenatal diagnosis and termination for anomaly. Health Care Women Int 2009;30:507–35. 19418323 10.1080/07399330902801278

[5] Lalor J , Begley CM , Galavan E . Recasting hope: A process of adaptation following fetal anomaly diagnosis. Soc Sci Med 2009;68:462–72. 19026477 10.1016/j.socscimed.2008.09.069

[6] Sandelowski M , Barroso J . The travesty of choosing after positive prenatal diagnosis. J Obstet Gynecol Neonatal Nurs 2005;34:307–18. 10.1177/0884217505276291 15890829

[7] Wool C . Systematic review of the literature: Parental outcomes after diagnosis of fetal anomaly. Adv Neonatal Care 2011;11:182–92. 21730912 10.1097/ANC.0b013e31821bd92d

[8] Kaasen A , Helbig A , Malt UF , *et al.* Acute maternal social dysfunction, health perception and psychological distress after ultrasonographic detection of a fetal structural anomaly. BJOG 2010;117:1127–38. 20528866 10.1111/j.1471-0528.2010.02622.x

[9] Rona RJ , Smeeton NC , Beech R , *et al.* Anxiety and depression in mothers related to severe malformation of the heart of the child and foetus. Acta Paediatr 1998;87:201–5. 9512209 10.1080/08035259850157679

[10] Howe D . Ethics of prenatal ultrasound. Best Pract Res Clin Obstet Gynaecol 2014;28:443–51. 24374013 10.1016/j.bpobgyn.2013.10.005

[11] Asplin N , Wessel H , Marions L , Georgsson Öhman S . Pregnant women's experiences, needs, and preferences regarding information about malformations detected by ultrasound scan. Sex Reprod Healthc 2012;3:73–8. 22578754 10.1016/j.srhc.2011.12.002

[12] Carlsson T , Bergman G , Melander Marttala U , Wadenste B , Mattsson E . Information following a diagnosis of congenital heart defect: experiences among parents to prenatally diagnosed children. PLoS One 2015;10:e0117995. 25692879 10.1371/journal.pone.0117995 PMC4333226

[13] Lalor JG , Begley CM , Galavan E . A grounded theory study of information preference and coping styles following antenatal diagnosis of foetal abnormality. J Adv Nurs 2008;64:185–94. 18990100 10.1111/j.1365-2648.2008.04778.x

[14] Arya B , Glickstein JS , Levasseur SM , Williams IA . Parents of children with congenital heart disease prefer more information than cardiologists provide. Congenit Heart Dis 2013;8:78–85. 22891764 10.1111/j.1747-0803.2012.00706.x PMC3502642

[15] Carlsson T , Bergman G , Karlsson AM , Mattsson E . Content and quality of information websites about congenital heart defects following a prenatal diagnosis. Interact J Med Res 2015;4:e4. 25608457 10.2196/ijmr.3819 PMC4319076

[16] Sveriges Riksdag . Abortlag 1974:595 [Abortion act 1974:595] [WWW document]. URL http://www.riksdagen.se/sv/Dokument‐Lagar/Lagar/Svenskforfattningssamling/Abortlag‐1974595_sfs‐1974‐595/ [accessed on 16 June 2015].

[17] Polit DF , Beck CT . Nursing Research: Generating and Assessing Evidence for Nursing Practice. Lippincott Williams & Wilkins: Philadelphia; 2012.

[18] Patton MQ . Qualitative Research & Evaluation Methods. Sage publications: California; 2002.

[19] Graneheim UH , Lundman B . Qualitative content analysis in nursing research: Concepts, procedures and measures to achieve trustworthiness. Nurse Educ Today 2004;24:105–12. 14769454 10.1016/j.nedt.2003.10.001

[20] Astbury‐Ward E , Parry O , Carnwell R . Stigma, abortion, and disclosure – Findings from a qualitative study. J Sex Med 2012;9:3137–47. 22239919 10.1111/j.1743-6109.2011.02604.x

[21] Shellenberg KM , Moore AM , Bankole A , *et al.* Social stigma and disclosure about induced abortion: Results from an exploratory study. Glob Public Health 2011;6(Suppl 1):S111–25. 21745033 10.1080/17441692.2011.594072

[22] Fox R , Evans K , Bale S , *et al.* Amniocentesis counselling: A role for the midwife‐practitioner. J Obstet Gynaecol 2008;28:189–93. 18393017 10.1080/01443610801912279

[23] Menahem S , Grimwade J . Counselling strategies in the prenatal diagnosis of major heart abnormality. Heart Lung Circ 2004;13:261–5. 16352205 10.1016/j.hlc.2004.06.009

[24] Atkinson RC , Shiffrin RM . Human memory: A proposed system and its control processes In The Psychology of Learning and Motivation, Spence KW, Spence JT (eds). New York: Academic press, 1968;89–195.

[25] Caldera K , Ha D , Menahem S . The development of a CD‐ROM: An aid to fetal cardiac diagnosis and counseling. Fetal Diagn Ther 2013;33:61–4. 22814118 10.1159/000339655

[26] Johnson A , Sandford J , Tyndall J . Written and verbal information versus verbal information only for patients being discharged from acute hospital settings to home. Cochrane Database Syst Rev 2003;4:CD003716. 14583990 10.1002/14651858.CD003716 PMC6991927

[27] Hilton‐Kamm D , Sklansky M , Chang RK . How not to tell parents about their child's new diagnosis of congenital heart disease: An Internet survey of 841 parents. Pediatr Cardiol 2014;35:239–52. 23925415 10.1007/s00246-013-0765-6

[28] Norvik G . Is there a bias against telephone interviews in qualitative research? Res Nurs Health 2008;31:391–8. 18203128 10.1002/nur.20259 PMC3238794

[29] Opdenakker R . Advantages and disadvantages of four interview techniques in qualitative research. Forum Qual Soc Res 2006;7:11.

[30] Sturges JE , Hantahan KJ . Comparing telephone and face‐to‐face qualitative interviewing: A research note. Qual Res 2004;4:107–18.

[31] Kavanaugh K , Ayres L . “Not as bad as it could have been”: Assessing and mitigating harm during research interviews on sensitive topics. Res Nurs Health 1998;21:91–7. 9472241 10.1002/(sici)1098-240x(199802)21:1<91::aid-nur10>3.0.co;2-c

[32] Irvine A . Duration, dominance and depth in telephone and face‐to‐face interviews: A comparative exploration. Int J Qual Methods 2011;10:202–20.

