# Supplementary material for: Experiences of informational needs and received information following a prenatal diagnosis of congenital heart defect
Source: Prenat Diagn. 2016 Apr 24;36(6):515–22. doi: 10.1002/pd.4815 (PMC5074242; doi:10.1002/pd.4815)
Supplement: Supplementary file 1 — Interview guide [file PD-36-515-s001.docx]

| **Topic** | **Main question** | **Sub-question** |
| --- | --- | --- |
| Informational needs | Can you describe what information you experienced that you needed when you received the diagnosis? | What topics do you think that it is particularly important to get information on at the time? |
|  |  | In what way would you have preferred to get information? |
| Information received | Can you describe how you experienced the information from the health professionals? | What information was particularly useful? |
|  |  | What information was missing? |
|  |  | Did you get answers to your questions? |
|  |  | Did you experience any information as superfluous? |
|  | Can you describe if and how you looked for information yourself? | What did you find? |
|  |  | How did you experience what you found? |
