# Supplementary material for: Experiences of informational needs and received information following a prenatal diagnosis of congenital heart defect
Source: Prenat Diagn. 2016 Apr 24;36(6):515–22. doi: 10.1002/pd.4815 (PMC5074242; doi:10.1002/pd.4815)
Supplement: Supplementary file 2 — Identified needs for informational methods with illustrative quotes [file PD-36-515-s002.docx]

| **Informational method needs** | **Continued pregnancy (N=11)** | **Terminated pregnancy (N=15)** |
| --- | --- | --- |
| Available | *So you don’t want to be a bother and then you think, I’ll wait until I have my appointment in two weeks. If you could just write the question and get it done with, you would probably do it like... (Female 1)* | *Though we got something which made things easier I think, we got a direct line to everyone. (Female 3)* |
| Detailed | *As much as possible and then I can kind of sort out what I, what I can take in. (Female 10)* | *You want to know everything in the greatest detail. (Male 10)* |
| Early | *Of course you want to find out about it as early as possible... (Male 7)* | *I cannot emphasize enough what a success factor I think it is for those people who are in this position to get fast answers. (Male 8)* |
| Honest | *And then, when you have heard both the bad and the good, then you don’t have, then it’s much…um… more difficult to have preconceived ideas. (Male 6)* | *It’s better that they say precisely what they think it is rather than not saying anything. (Female 4)* |
| Illustrations | *Be a little pedagogic as well, draw a heart on a piece of paper and explain how the heart works and how things could turn out and… (Male 7)* | *It became very clear when he drew it and explained things using the picture he drew. So.. it gives you another understanding.. (Female 14)* |
| Mother tongue | *What could have been better, that we could have got it [an illustration] in Swedish. (Female 7)* | *Yes, perhaps it would be good. Bearing in mind that Spanish is a big language as well. (Male 2)* |
| Objective | *It should probably still be very, like, yes very factual.*  *(Male 1)* | *If someone had sort of... If someone had been for it [one of the alternatives]... then you couldn’t have made a choice. (Female 4)* |
| Specialist information | *If you talk to a doctor who is a specialist in the field, then you want to have information from so close to the source as possible (Male 6)* | *Personally I would rather have got the information from the Swedish health service, as I feel I trust that they know what they’re talking about.*  *(Female 6)* |
| Repeated | *Perhaps you could have a later meeting with a heart specialist.*  *(Female 2)* | *Then we met them several times as well, so then we remembered everything. (Female 3)* |
| Written | *And therefore this need for a brochure or an Internet site or something where you can go back and read… (Female 9)* | *If you had had an information leaflet, perhaps in Swedish, that you could have taken home, that would have been very much appreciated. (Female 3)* |
