# Supplementary material for: Experiences of informational needs and received information following a prenatal diagnosis of congenital heart defect
Source: Prenat Diagn. 2016 Apr 24;36(6):515–22. doi: 10.1002/pd.4815 (PMC5074242; doi:10.1002/pd.4815)
Supplement: Supplementary file 3 — Identified needs for informational content with illustrative quotes [file PD-36-515-s003.docx]

| **Content** | | | | **Continued of pregnancy (N=11)** | **Termination of pregnancy (N=15)** |  |  |
| --- | --- | --- | --- | --- | --- | --- | --- |
| Anatomy | |  | | *The most important thing was to know what the actual heart defect looked like. (Female 7)* | *What it looks like and what happens and what it leads to, that information was invaluable. (Female 5)* |  |  |
| Associated anomalies | |  | | *Tendency in children with Down’s syndrome for example to have that kind of heart defect. (Female 10)* | *It was all that about Down’s syndrome. (Male 8)* |  |  |
| Causes | |  | | *Not mentioned* | *We asked, tried to find out... is it our lifestyle or what we have done. (Male 5)* |  |  |
| Termination of pregnancy | | | | |  |  |  |
|  | Fetal care | | *Not mentioned* | | *I would gladly have got a bit more information about how the fetus is taken care of. (Female 6)* |  |  |
|  | Fetal status | | *Not mentioned* | | *Yes kind of, does the child suffer... Yes, but you think a lot about things like that.. (Female 12)* |  |  |
|  | Recuperation | | *Not mentioned* | | *Can I work out again, is it OK? (Female 3)* |  |  |
|  | Decision to see the fetus | | *Not mentioned* | | *When you say that we want to terminate. And then we signed a lot of papers and those things, I think you can go in there and say that this is how it is, when the fetus has come out, you will have the chance to, yes, see the child or fetus. (Male 8)* |  |  |
|  | The procedure | | *Not mentioned* | | *It can be extra important that they explain how a termination is done. (Female 6)* |  |  |
|  | The option | | *What choices you can make. Um, and it was good to hear. (Male 11)* | | *That we needed to decide whether we wanted to continue the pregnancy or not. (Female 3)* |  |  |
|  | Time required | | Not mentioned | | *We needed to fix a babysitter and so it might have been better if we were told that it would be such a long time... (Male 3)* |  |  |
| Postnatal situation | | | |  |  | | |
|  | Activity | | *If she could go out in the woods with us and do that kind of thing. (Male 6)* | | *How a person is affected, does it kind of have a sporting chance of yes, being activated . (Male 8)* | |  |
|  | Family life | | *For the whole family like. (Female 8)* | | *Not mentioned* | |  |
|  | Follow-up care | | *Very important to know what the chain will look like.*  *(Male 1)* | | *Not mentioned* | |  |
|  | Mortality | | *What I thought about first was that we could lose one of the children. (Male 9)* | | *I wanted to know if he could live afterwards. That was kind of what was important. (Female 4)* | |  |
|  | Possible complications | | *What sort of problems the heart defect could entail.*  *(Female 7)* | | *Complications, I went home and Googled it. (Female 4)* | |  |
|  | Practical issues | | *You can’t go in the same transportation. (Female 2)* | | *As for example in Stockholm when it’s a question of whether the child must be operated on... that is that it must have a heart operation fairly soon after birth as we belong to Lund... So if you don’t know about it before and so on... (Female 6)* | |  |
|  | Prognosis | | *What the heart defect prognosis was, with the heart defect. (Female 7)* | | *What is the prognosis? What happens if we decide to continue?*  *(Male 12)* | |  |
|  | Quality of life for the child | | That it can go and have a good life. (Female 8) | | *How it would then be for the child to live with the heart defect.*  *(Female 11)* | |  |
|  | School | | *If she could go to school as normal. (Male 6)* | | *Not mentioned* | |  |
|  | Treatments | | *If it is kind of possible.... to correct this problem. (Male 1)* | | *If it was somehow possible to operate. (Female 11)* | |  |
| Previous cases | | | | *Listen to other people who have gone through exactly the same thing is quite... I like that. (Male 11)* | *How things work for them, what happened to their relationship afterwards, do they struggle on like, have they got a new one.*  *(Male 10)* | | |
| Professional psychosocial support | | | | *Not mentioned* | *She asked me why don’t you talk to someone in the meantime, and I just, I didn’t know that it was even possible. (Female 6)* | | |
| Statistics | | | | *When I see statistics that they, there are very many children who survive, it would be very reassuring. (Male 9)* | *In a perfect world or what should I say, I would have liked statistics, if you know what I mean. (Female 6)* | | |
